# Deprotonated of layered double hydroxides during electrocatalytic water oxidation for multi‐cations intercalation

**DOI:** 10.1002/smo.20230026

**Published:** 2024-04-10

**Authors:** Bowen Jin, Jianxiong Gao, Yunqi Zhang, Mingfei Shao

**Affiliations:** ^1^ State Key Laboratory of Chemical Resource Engineering Beijing University of Chemical Technology Beijing China; ^2^ Renewable Energy Research Institute Quzhou Institute for Innovation in Resource Chemical Engineering Quzhou China

**Keywords:** deprotonation, energy storage, layered double hydroxides, multi‐ion battery

## Abstract

Aqueous rechargeable batteries using abundant multi‐ion cations have received increasing attention in the energy storage field for their high safety and low cost. Layered double hydroxides (LDHs) possess a two‐dimensional structure and exhibit great potential as cathodes for multi‐ion intercalation. However, the insufficient active sites of LDHs result in low capacities in the discharging process. Interestingly, the LDHs after the deprotonation process exhibit favorable electrochemical performance of multi‐cation intercalation. The deprotonation process of LDHs has been widely found in the oxygen evolution reaction and energy storage field, where LDHs lose H in laminates and converts to deprotonated *γ*‐phase MOOHs (MOOs). Herein, we take a comprehensive overview of the dynamics structure transformation of the deprotonation process of LDHs. Furthermore, the development of advanced aqueous battery cathode and metal battery anode based on deprotonated LDHs for energy storage is explored and summarized. Finally, the perspective of deprotonated LDHs in the energy storage field is discussed.

## Graphical abstract

The deprotonated LDHs exhibit high performance for multi‐ion intercalation. In this review, we focus on the dynamics structure transformation of the deprotonation process of LDHs. We then summarize the status and issues of deprotonated LDHs for advanced aqueous battery cathode and metal battery anode. Finally, the future development perspectives of deprotonated LDHs in the energy storage field are prospected.

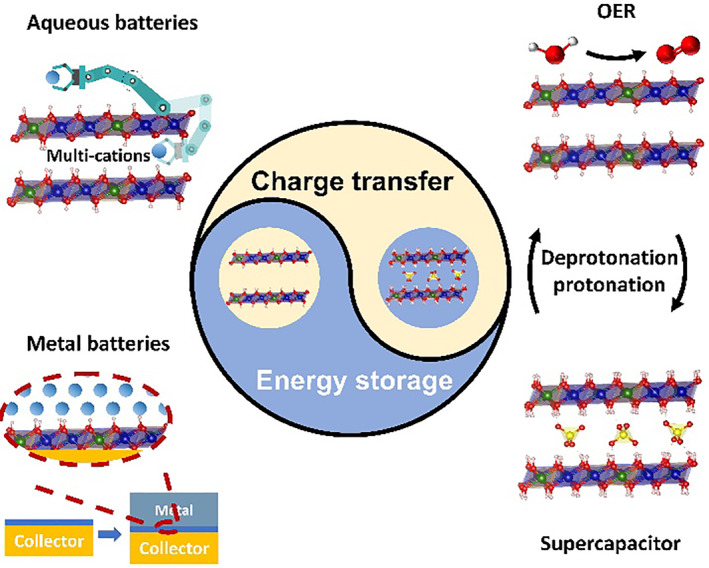

## 1 INTRODUCTION

The development of renewable energy storage devices is receiving increasing attention due to the growth of future energy requirement.^[1]^ One crucial element in energy management devices are Li ion batteries that provide high energy/power density and long service life. However, to meet the high demand of high safety and low cost for advanced portable devices, batteries face new challenges and requirements. An alternative to Li ion batteries is multi‐cation aqueous batteries that may also provide high capacity but additionally have the advantage of both aqueous battery and multi‐cation battery. Aqueous batteries are intrinsically safe for its nonflammable, nontoxic of “water” solvate.[^2^, ^3^, ^4^] Abundant and non‐toxic multi‐ion cations, for example, Na^+^, K^+^, Ca^2+^, Mg^2+^ offer better opportunities for large‐scale applications.[^5^, ^6^] Despite continuous progress, only few materials (*e.g.,* MXene^[7]^, MoS_2_ [^8^, ^9^], MoO_*x*_ ^[10]^, Prussian blue analogue^[11]^ and organic polymers[^12^, ^13^]) have been successfully explored for reversible multiple‐cation intercalation. Unfortunately, these materials either performed as anode for cation intercalation or exhibits low theorical capacity in positive potential window, which means these materials cannot work as cathodes for various cations storage. Therefore, it is highly desired to develop a positive electrode with high performance for multi‐cation intercalation.

LDHs, are a series of two‐dimensional nanomaterials, whose structure is based on well‐defined interlayer multi‐metal cations and interlayer anions.^[14]^ The general formula of LDHs is expressed by [M_1−*x*_^2+^M_*x*_^3+^(OH)_2_]_*x*_^+^(A^n−^)_*x*_/*m*·*n*H_2_O, where M^2+^, M^3+^ representing divalent metal ions (*e.g.* Mg^2+^, Ni^2+^, Zn^2+^, Co^2+^, etc.) and trivalent metal ions (such as Al^3+^, Fe^3+^, V^3+^, Co^3+^, Mn^3+^) respectively, A^*n*−^ expressing anions (including inorganic anions (such as F^−^, Cl^−^, Br^−^, SO_4_^2−^), organic anions (acetate, lactate, dodecyl sulfate) or polyacid anions, organic molecules), *x* is the molar ratio of M^3+^/(M^2+^+M^3+^), which usually varies between 0.2 and 0.4 for structural stability factors. The host layers and the guest interlayers with different charges are stacked alternately through non‐covalent interactions (*e.g.,* electrostatic interaction, hydrogen bonding and van der Waals force *etc.*). Benefitting from their unique structure, LDHs are considered promising for use in the electrochemical field (*e.g.* electrocatalytic oxidation and energy storage) for its high electrochemical activity.[^15^, ^16^, ^17^] Especially, LDHs show similar OER electrocatalytic activities with the best precious metal‐based electrocatalysts (*e.g.,* IrO_2_).^[18]^ Moreover, the LDHs have also been considered as supercapacitor electrode materials due to their high redox activities. Interestingly, both these processes (OER and supercapacitor) involve the phase transformation (deprotonation) process of LDHs.^[19]^ Specifically, OH^−^ adsorbed on the surface of LDHs and promotes breaking the O–H bond on LDHs. Proton(s) H in LDH laminate is lost (deprotonation) and LDHs converts to deprotonated LDHs (MOOHs or MOOs). The capacitive behaviors of LDHs are attributed to the redox reaction within the deprotonation process. As for the OER process, LDHs first converts to MOOHs (MOOs), then the deprotonated LDHs electrooxidize water to release O_2_. Besides, the deprotonated LDHs have also been found to be an aqueous battery cathode in our recent work.^[20]^ The deprotonated LDHs exhibit negative adsorption energies toward various cations, which exhibit great potential for hosting multi‐cation intercalation. High capacity of 156, 151, 198, 172, 217, and 136 mAh g^−1^ is achieved by deprotonated CoFe‐LDH for Li^+^, Na^+^, K^+^, Ca^2+^, Mg^2+^, and Zn^2+^ intercalation, which is 27, 27, 33, 34, 43, and 24 times larger than that of pure CoFe‐LDH without deprotonation process, respectively. This work pioneers a new avenue for the application of LDHs for energy storage.

**FIGURE 1:**
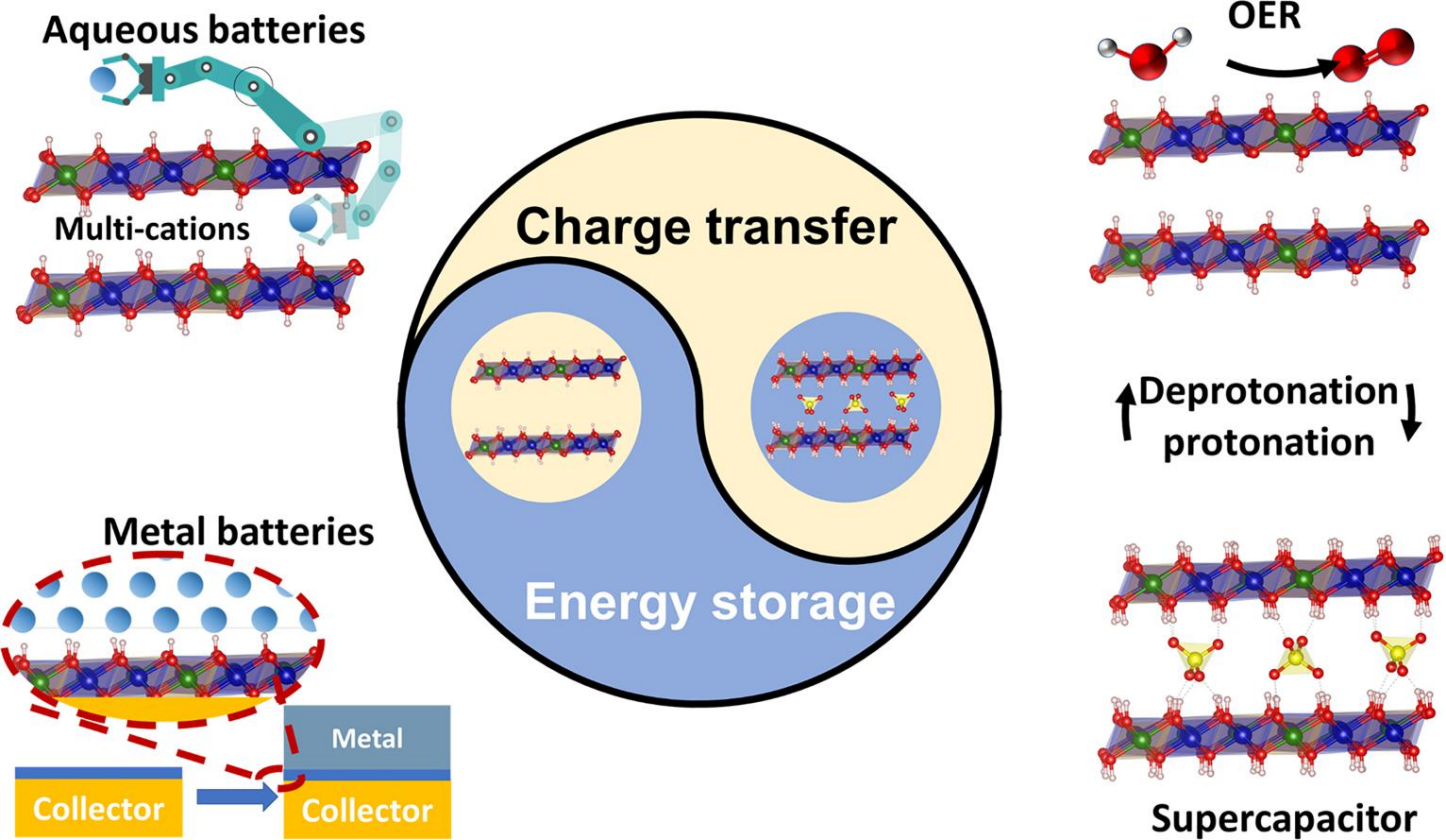
Schematic illustration of the application of deprotonated LDHs.

Understanding the dynamics deprotonation process of LDHs in electrochemical processes is expected to develop advanced battery electrode materials with high‐performance. Unfortunately, few reports focus on the phase transformation process of LDHs. In this review, we bridge the gap and provide a comprehensive perspective of deprotonated LDHs in the electrochemical field (Figure 1). Furthermore, the development of advanced aqueous battery cathode and metal battery anode based on deprotonated LDHs for energy storage is explored and summarized. Finally, we offer perspectives into the challenges faced by deprotonated LDHs in the energy storage field.

## 2 THE DEPROTONATION PROCESS OF LDHS

**FIGURE 2:**
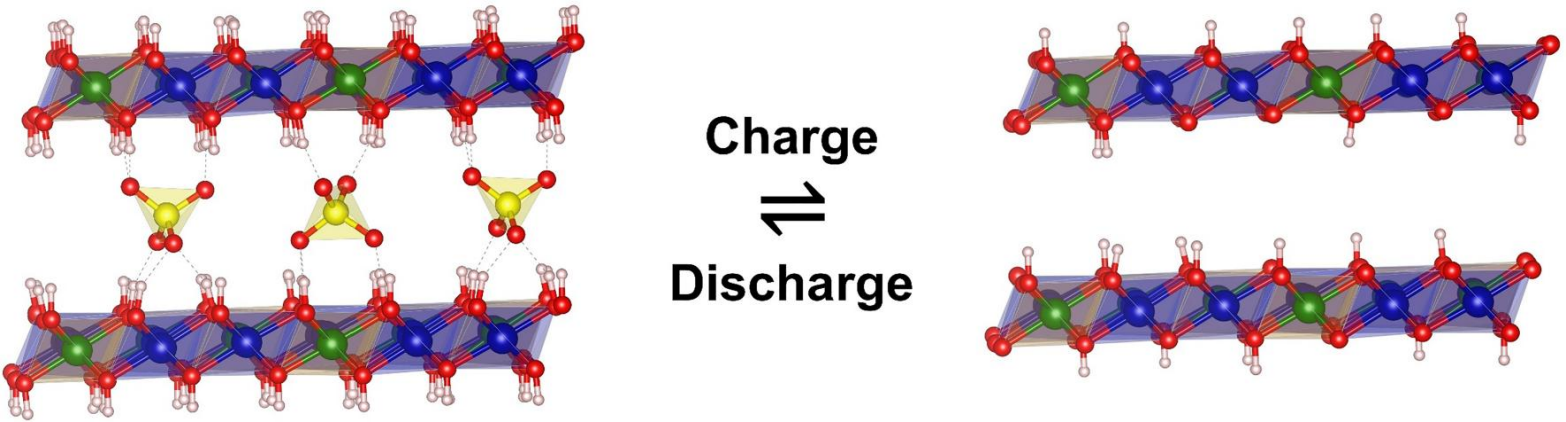
Schematic demonstrating the charging/discharging process of LDH supercapacitor cathodes.

The deprotonation process of LDHs has been widely found in supercapacitors and OERs in recent years. Supercapacitors, known as electrochemical capacitors, are promising energy storage devices due to their high safety, high power and extremely long cycle life.[^21^, ^22^, ^23^] The energy‐storage mechanism of supercapacitor electrodes can be classified as electric double‐layer capacitors (EDLC) and pseudocapacitors.^[23]^ The pseudocapacitive materials store energy via reversible Faradaic redox reactions at the surface, subsurface (RuO_2_, MnO_2_ *etc.*)^[24]^ or bulk (Nb_2_O_5_, MoO_3_ *etc.*)[^25^, ^26^] of active materials, which provides higher capacitance than EDLC. LDHs with variable valence metal elements for example, Ni‐LDH, Co‐LDH, Mn‐LDH are widely investigated as positive pseudocapacitive materials.^[27]^ The charging process of LDHs in the supercapacitor cathode is essentially the deprotonation process of LDHs. Figure 2 gives an example of the chemical/crystal structure of Co based LDH (CoAl‐LDH) during the charging/discharging (deprotonation/protonation) process. The pseudocapacitance mechanism of LDHs is shown as follows:

(1) $$\text{Co}{\left(\text{OH}\right)}_{2}+{\text{OH}}^{-}↔\text{CoOOH}+H_{2}O+e^{-}$$

(2) $$\text{CoOOH}+{\text{OH}}^{-}↔\text{Co}O_{2}+H_{2}O+e^{-}$$

CoAl‐LDH consist of Co(OH)_6_ octahedron unit, where each ‐OH group is shared by three adjacent octahedral units. The active composition of CoAl‐LDH is the same as Co(OH)_2_ in nature. The charging process of Co‐based LDH is divided into two steps. Proton H in Co(OH)_2_ laminate is first lost (deprotonation) and releases an electron. In the process, *γ*‐CoOOH species was converted by Co(OH)_2_. In the second step, the residual proton H in the laminate lost and released an electron after reacting with OH^−^ and formats the CoO_2_ species. The CoAl‐LDH supercapacitor electrode achieved electrochemical energy storage through the valence variation of Co with reversible adsorption and desorption of proton H on the laminates. Mousty ’s group investigated the charging/discharging mechanism of CoAl‐LDH via the Raman spectrum.^[28]^ The stable *γ*‐Co_1−*x*_Al_*x*_OOH phase is irreversibly oxidized by CoAl‐LDH and most of the Co element is oxidized to Co^3+^ during the first oxidative CV scan. Two pairs of redox peaks can be observed in the CV measurement, which are assigned to the transformation of Co^2+^/Co^3+^ and Co^3+^/Co^4+^ ions, respectively. These studies provide evidence for the pseudocapacitance mechanism with the reversible protonation/deprotonation on LDH laminates in the electrochemical redox process (Equations (1) and (2)), that is, the energy storage process of LDHs can be expressed by the breaking or forming of the O–H bond and metal element redox.

**FIGURE 3:**
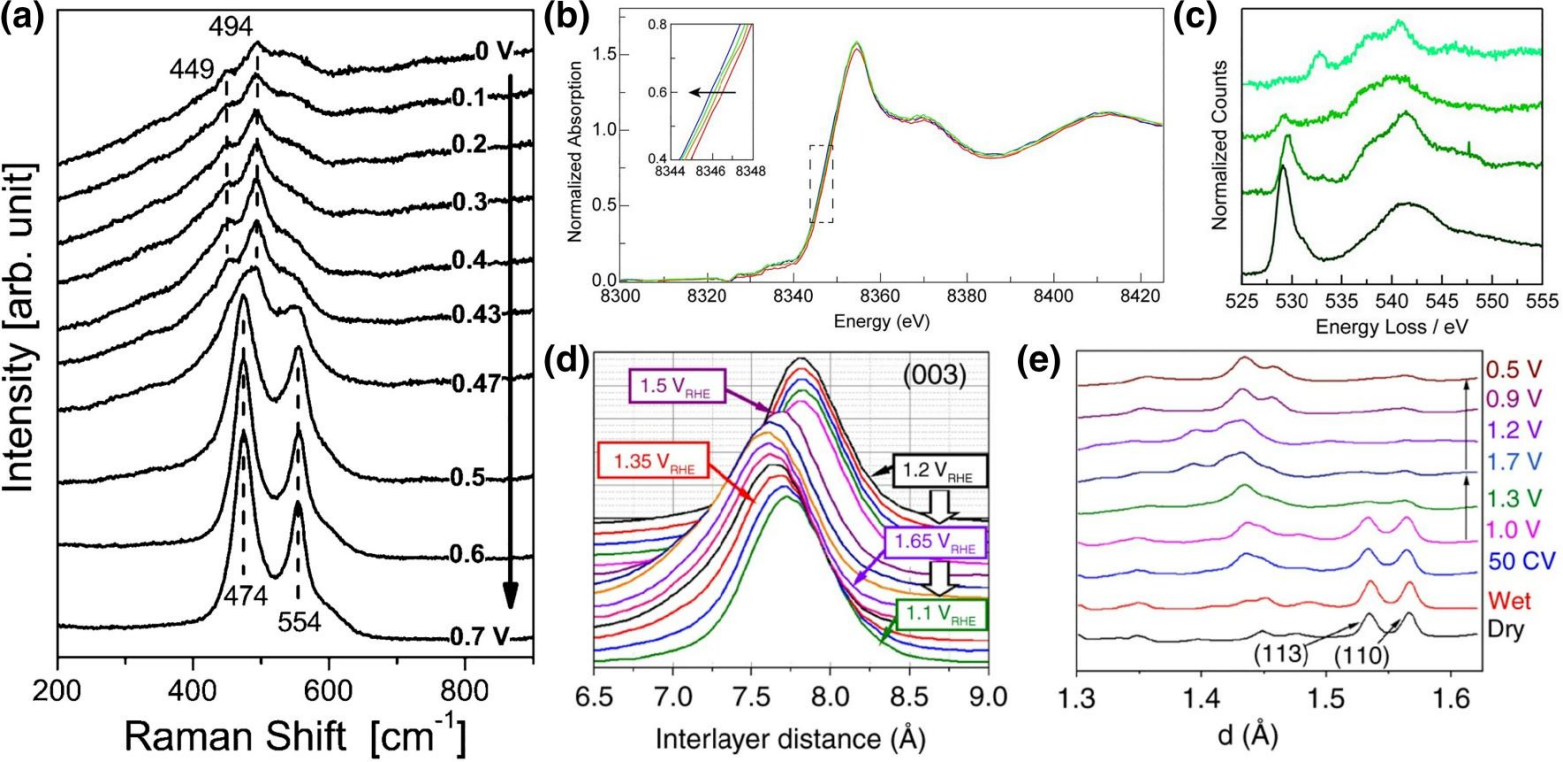
(a) In situ Raman spectra of Ni film deposited Au substrate in 0.1 M KOH versus Hg/HgO at different potential window.^[31]^ *Copyright © 2013 American Chemical Society*. (b) Ni K‐edge XANES spectra of Ni based hydroxide with 12.30 moL % Fe (blue), 0.58 moL % Fe (red), 2.66 moL % Fe (orange), and 6.45 moL % Fe (green). (c) Oxygen K‐edge EELS of nickel oxide model compounds. From top to bottom: Ni^II^O, Ni^II^(OH)_2_, LiNi^III^O_2_, and *γ*‐Ni^III/IV^OOH.^[32]^ *Copyright © 2017 National Academy of Sciences.* (d) Waterfall plots of normalized and background‐subtracted (003) peaks obtained during in situ WAXS and potential steps for NiFe‐LDH in 0.1 M KOH.^[34]^ (e) in situ WAXS patterns for d‐values close to the (110) peak of NiFe LDH under various conditions.^[34]^ *Copyright © 2020 Springer Nature Limited.*

The deprotonation process of LDHs can also be found in the OER process. OER is an anodic half‐cell reaction water electrolysis, which involves sluggish four‐electron transfer with OH^−^ coupling processes.^[29]^ The first 3 d transition metals LDHs are potential alternatives in the electrocatalytic water oxidation field for their intriguing intrinsic activity. Various 3 d iron group metals (Fe, Co and Ni) based LDHs for example, Ni‐Fe, Ni‐Co, Co‐Ni, Fe‐Ni, and Fe‐Co, have been investigated as electrocatalysts to achieve OER with a high efficiency.^[30]^ Interestingly, the OER process is normally accompanied by redox reactions and phase transformation of LDHs, especially for NiFe‐LDH. These processes (redox reactions prior to OER) is similar as charging process in supercapacitor, that is the deprotonation. Bell's group investigated the surface transformation of NiFe‐LDH via in situ Raman spectra, as shown in Figure 3a. Two obvious peaks are identified in the range of 445–465 cm^−1^ for pristine NiFe‐LDH, which represents the Ni–O vibration of Ni(OH)_2_. Pairs of new bands representing the Ni–O vibrations of NiOOH at 474 and 554 cm^−1^ appear with increasing the potential to 0.47 V and above, indicating phase transformation of Ni(OH)_2_ to NiOOH (deprotonation process) before OER process.^[31]^ Also, Nocera's group revealed that the NiFe‐LDH mediated OER is Ni^4+^‐containing *γ*‐NiOOH phase by in situ Ni K‐edge XAS (Figure 3b) and O K‐edge electron energy loss spectroscopy (EELS, Figure 3c).^[32]^ Not only the valence of Ni species but also Fe species in LDHs increases with increasing applied potential window to OER. Sun's group reported that the valence states of Fe ions in NiFe‐LDH without applied potential are mixed both +2 and +3. The iron's valance in Fe^2+^‐NiFe‐LDH increased to +3.22 at 1.5 V versus RHE, demonstrating that partial Fe^4+^ among all Fe species are detected during OER. More interestingly, the high‐valence of Fe^(3+δ)+^ species is still present even when the potential switch back to 0 V, that is, the phase transformation of LDHs is irreversible. Stahl's group also confirmed the high‐valence of Fe^(3+δ)+^ species in the NiFe‐LDH catalyst during steady‐state water oxidation via Mössbauer spectra.^[33]^ A doublet with an isomer shift (*δ*) of 0.34 mm/s and quadrupole splitting (Δ) of 0.46 mm/s of the NiFe catalyst was exhibited in the initial Mössbauer spectra under open‐circuit conditions. A shoulder spectrum at *δ* = −0.27 mm/s appeared at a high potential of 1.62 V, reflecting the high oxidation Fe sites (approximately 12%) in the material. The intensity of oxidized Fe peak is increased with applied potential, accounting for approximately 21% of the total Fe oxidized to Fe^4+^ at the potential of 1.76 V during steady‐state catalysis. Moreover, the oxidized Fe species (Fe^4+^, ∼20% of total Fe) was still detected, when the potential was returned to 1.49 V. Strasser’s group investigated the phase transition of CoFe‐LDH (and NiFe‐LDH) at a high potential range (OER process).^[34]^ *Operando* wide‐angle X‐ray scattering (WAXS) and X‐ray absorption spectroscopy (XAS), as well as synergistic DFT results revealed that CoFe LDHs with *α*‐phase undergo phase transformation to a deprotonated *γ*‐phase. ∼8% contractions on both interlayer distances (from 7.7 Å to 7.1 Å) and in‐plane metal–metal distances (from 3.1 Å to 2.85 Å) are characterized upon the phase transition, which are induced by the oxidation of Fe and Co (Ni) to a high valence state at the potential above the oxidation peak (Figure 3d,e). Concurrently, the interlayer carbonate anions are expelled, and K^+^ cations from the KOH electrolyte are intercalated with interlayer water still present in *γ*‐phase. The oxidation of CoFe and NiFe‐LDH results in a *γ*‐phase oxy‐hydroxide that has a stoichiometry of K_1/4_(H_2_O)_1/2_MO_2_.^[35]^ Similar to the OER process, the organic electrooxidation process also occurred on the anode. Both processes involve the oxygen species transformation, which probably determines the directions of electrochemical oxidation when operated in a mixture medium. In this context, LDHs have received great interests as advanced electrocatalysts due to their advantages of high activity and cost‐effectiveness. Similarly, the deprotonated process of LDHs has also been found in organic electrooxidation processes. The hydroxy groups on the host layer of LDHs are easily deprotonated at low voltage before organic electrooxidation. The exposed oxygen sites formed in LDHs can oxidize the organic molecules.[^15^, ^36^, ^37^, ^38^, ^39^, ^40^]

**FIGURE 4:**
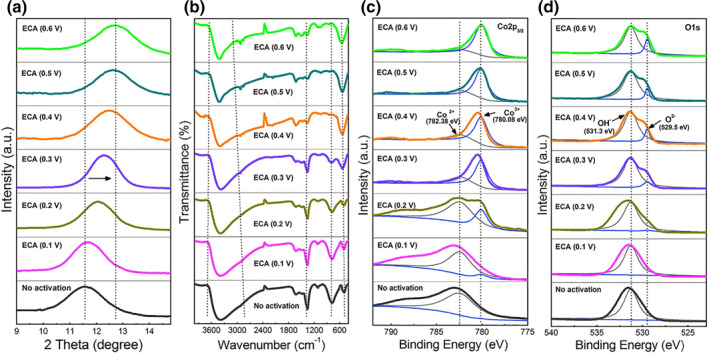
Structure and composition changes of CoFe‐LDH after different deprotonation processes: (a) XRD patterns of the (003) reflection of CoFe‐LDH with different deprotonation processes. FTIR spectra (b), high‐resolution Co 2p3/2 XPS spectra (c), and high‐resolution O 1s XPS spectra (d) of deprotonated CoFe‐LDH with different deprotonation processes.^[20]^ *Copyright © 2018 Cell Press.*

Recently, an irreversible deprotonation process of Co‐based LDHs (*e.g.*, CoFe‐LDH, CoNi‐LDH and CoAl‐LDH) was investigated by our group.^[20]^ The deprotonated LDHs were formatted using a facile cyclic voltammetry method with different operation potential windows in an alkali electrolyte. The CV curve area of CoFe‐LDH was enlarged gradually with increasing potential window until 0.6 V versus SCE and finally reached a stable level after few cycles, indicating the irreversible deprotonation process. In this process, this deprotonated process is accompanied with the decreasing of CoNi‐LDH interlayer distance, which is attributed to the reduction of the hydroxyl group in the CoFe‐LDH host layers and the loss of the interlayer (Figure 4a). Raman spectra show that a stretching band of Co–OH (798 cm^−1^) is vanished gradually and O–Co–O vibration at 568 cm^−1^ is emerged (Figure 4b). A new peak at 780.08 eV attributed to Co^3+^ arose after the deprotonation process in XPS measurements. Moreover, the intensity enhanced gradually with the deprotonation process, the corresponding concentration of Co^3+^ on LDH surface is increased from 0.4 wt% to 92.1 wt% after deprotonation (Figure 4c). The O^2‒^ species was also observed with increasing intensity after deprotonation. Co^3+^OOH in deprotonated LDH was stable even after a long cycling test, indicating the transformation of Co in CoFe‐LDH from Co(OH)_2_ to CoOOH in the deprotonation process (Figure 4d). However, the valence state of Fe remains Fe(III) oxidation state in this process. Benefiting from the “H vacancy” induced by Co^3+^, active oxygen species on deprotonated LDHs are exposed to adsorb various cations, representing the thermodynamically favorable process. The charging‐discharging electrochemical reaction is described as follows:

$$\left(\text{charge}\right)\;\left[{\text{Co}}^{3+}{\text{Fe}}_{0.5}^{3+}O_{1.5}{\left(\text{OH}\right)}_{1.5}\right]\cdot nH_{2}O+\left(\frac{y}{z}\right)M^{z+}+ye^{-}↔{\left[{\text{Co}}^{3-y}{{\text{Fe}}^{3+}}_{0.5}O_{1.5}{\left(\text{OH}\right)}_{1.5}\right]}^{-y}{\left(M^{z+}\right)}_{\frac{y}{z}}\cdot nH_{2}O\;\left(0<y<1\right)\;\left(\text{discharge}\right)$$

These results are in good line with Zhong's work.^[41]^ An irreversible oxidization reaction of Co^2+^ to Co^3+^ was found at the electrode interface in the first anodic polarization (prior to OER process). A broad and distinctive oxidation wave is observed for CV measurements in the potential of 1.1–1.3 V versus RHE on the first anodic process. No obvious reduction peak on the reverse sweep process even at a potential less than 1.2 V, indicating the irreversible interface reconstruction of Co^II^(OH)_2_ to Co^III^OOH. In the potential region higher than 1.43 V, a minor anodic peak corresponding to the oxidation reaction from Co^III^OOH to Co^IV^O_2_ is observed.^[41]^ Qiu group focus on the electronic and geometric structural evolution of Co based LDH (CoNi‐LDH) during the deprotonation process in CV measurements by combining operando X‐ray absorption fine structure (XAFs), ex‐situ soft X‐ray absorption spectroscopy (XAS) and synchrotron X‐ray diffraction (SXRD).^[42]^ The integrated area as well as redox peak intensity of CV curves increases with cycling number. The *Operando* X‐ray absorption near‐edge structure (XANES) spectra exhibit shifts of Co K‐edge position toward high energy in this process, indicative of the increasing Co oxidation state. The valence state of Co cations is stabilized after 110 cycles, which is in good agreement with the XAS results. The intensity of the Co L3‐edge at 779.6 eV is depressed and then remains stable with increasing the cycle number, while the high‐energy shoulder at 781.4 eV is increased and then remains stable. Finally, the portion of Co^3+^ ions in LDHs increases from 44% to 66%, indicting the partial conversion of Co(OH)_2_ to CoOOH in the deprotonation process. The portion of Ni^2+^ ions and the ratio of Ni^2+^ to Ni^3+^ exhibit negligible variation in the cycling, suggesting that the only Co sites LDH undergo a valence‐state and structural change into a high oxidation state with an unsaturated coordination structure.

**FIGURE 5:**
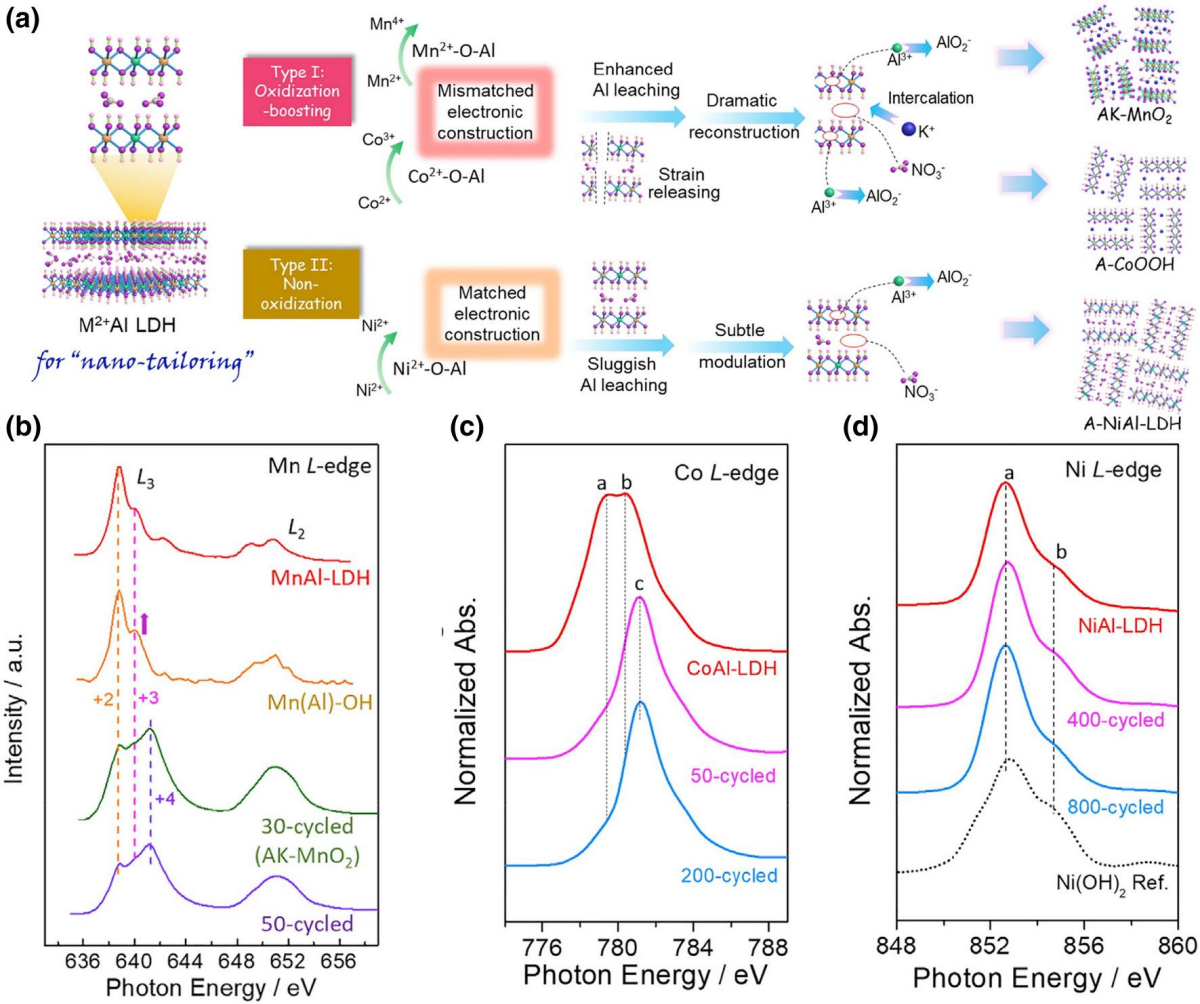
(a) Schematic illustration of the deprotonation process of MnAl, CoAl and NiAl‐LDH. EELS scans of Mn L‐edge in MnAl‐LDH (b), Co L‐edge in CoAl‐LDH (c) and Ni L‐edge in NiAl‐LDH (d) during cycling.^[43]^ *Copyright © 2021 Cell Press.*

Qiu’s group investigated the deprotonation process of different kinds of LDHs.^[43]^ Partly H (less 50%) in Co based LDH or Ni based LDH loss in the deprotonation process (Figure 5a). However, almost all the proton in the LDH laminate can be removed and releases two electron MnAl‐LDH in the deprotonation process. After cycling in an alkaline solution, the interlayer spacing of MnAl‐LDH increased from 4.7 Å to 7.0 Å with K^+^ intercalation. Mn L‐edge soft XAS spectra show the dominance of Mn^4+^ ions in deprotonated MnAl‐LDH, indicating the removal of most proton in the laminate. In this process, the partial Al is leached into the solution, resulting in the decreasing size of deprotonated LDHs (13 times smaller than pristine LDHs).

**FIGURE 6:**
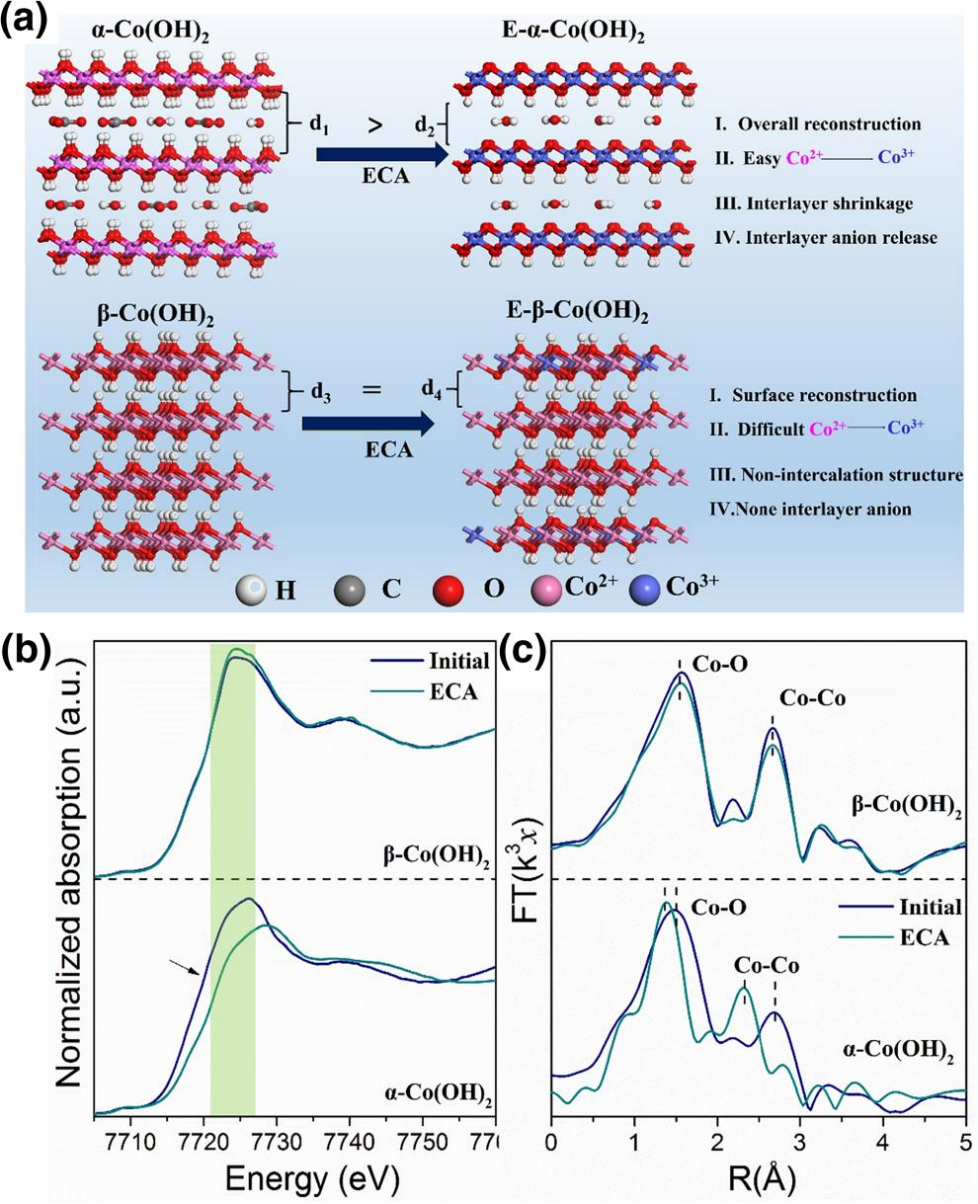
(a) Schematic illustration of the structure reconstruction of *α*‐and *β*‐Co(OH)_2_ after the deprotonation process. (b) Co Kedge XANES spectra and (c) the corresponding K2‐weighted Fourier transforms of Co K‐edge EXAFS spectra.^[44]^ *Copyright © 2021 Royal Society of Chemistry.*

Besides, other hydroxides with similar crystal phases also exhibit the similar deprotonation process of LDHs. We present 2D cobalt hydroxides with similar morphologies but different *α*‐ and *β*‐crystal phase structures.^[44]^ *α*‐Co(OH)_2_ shows large interlayer spacing (∼0.78 nm) with anion intercalation, while *β*‐Co(OH)_2_ possesses a narrow interlayer spacing of 0.31 nm without anion insertion. Interestingly, only *α*‐Co(OH)_2_ exhibits deprotonation process, where the interlayer spacing is decreased with interlayer anions releasing (Figure 6a). Moreover, the initial Co–OH bending vibration vanished and an O–Co–O vibration appeared in the Raman spectra. The EXAFS spectra exhibit an increase in the Co valence from +2 to +3, indicating the conversion of *α*‐Co(OH)_2_ to CoOOH in an alkaline solution during deprotonation. In contrast, the interlayer spacing, the interlayer anions and the composition of *β*‐Co(OH)_2_ were hardly activated and deprotonation (Figure 6b,c).

**FIGURE 7:**
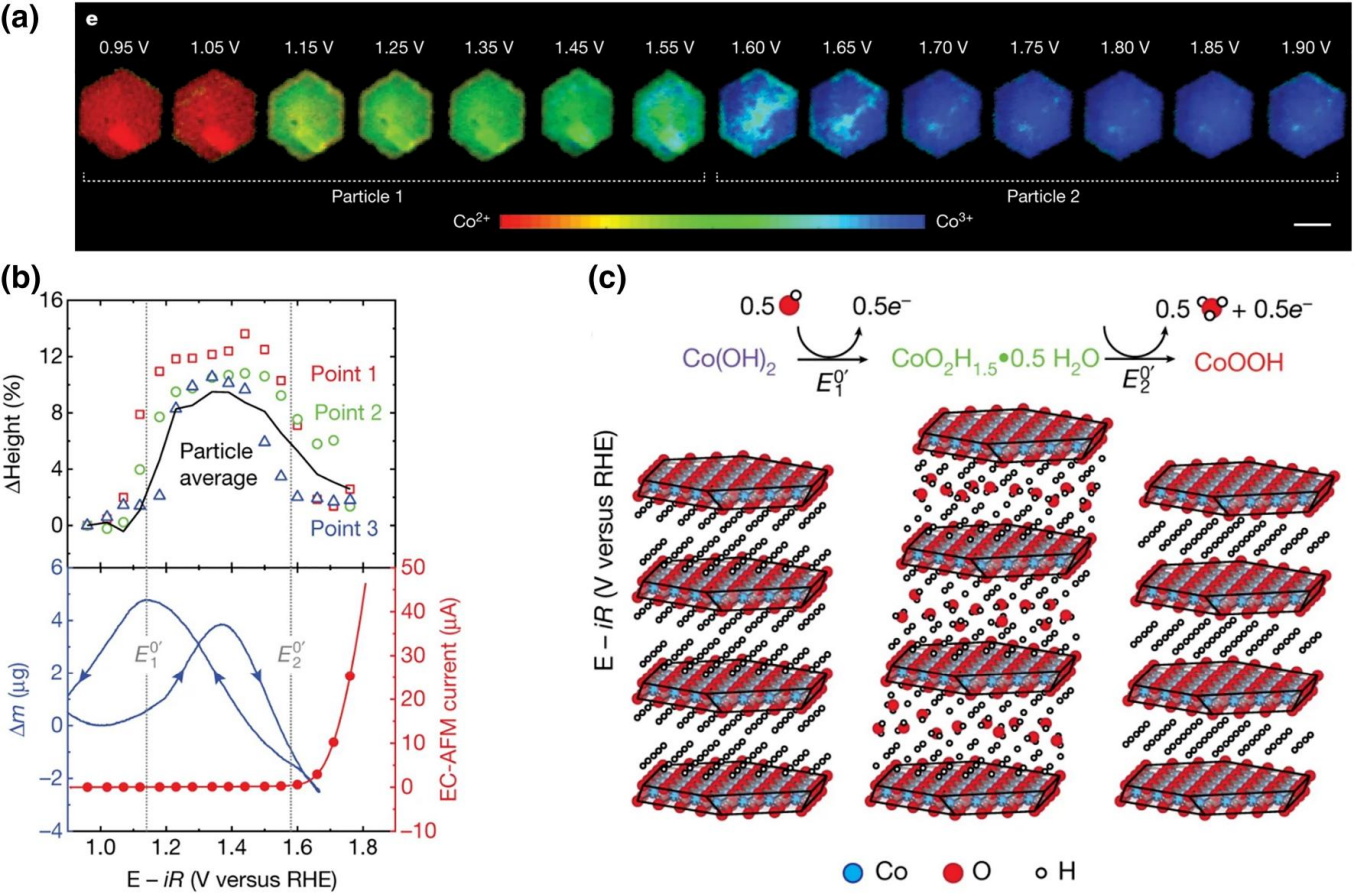
(a) Steady‐state voltage‐dependent Co oxidation state phase maps of *β*‐Co(OH)_2_ particles 1 and 2. Scale bar, 1 μm. (b) Top, Heights measured at three selected points (shown on the particle at 0.96 V in b); bottom, EC‐AFM current (linear sweep voltammetry, red line) and isothermal (*T* = 25 °C) mass change (measured by EQCM) with changing voltage (blue line). (c) Schematic demonstrating the translation and the expansion and contraction of individual CoO_2_ layers as the voltage is increased.^[29]^ *Copyright © 2021 Springer Nature Limited.*

William C. Chueh's group studied the phase transition of *β*‐Co(OH)_2_ prior to OER.^[29]^ The two oxidation peaks at 1.20 and 1.55 V *vs*. RHE, which have previously been attributed to the conversion of Co(OH)_2_ to CoOOH and of CoOOH to CoO_2_, are re‐investigated by electrochemical quartz crystal microbalance (EQCM) and operando scanning transmission X‐ray microscopy (STXM) (Figure 7). The results show that at the first oxidation peak, *β*‐Co(OH)_2_ deprotonated 0.5 H^+^, accompanied with 0.5 OH^−^ intercalation, to form *α*‐CoO_2_H_1.5_·0.5H_2_O that has interlayer water and +2.5 valance for Co. Then, at the second oxidation peak, *α*‐CoO_2_H_1.5_·0.5H_2_O is oxidized to *β*‐CoOOH that has no interlayer water, and the Co stays at +3 valence even at the OER potential, with no Co^4+^ observed.

## 3 DEPROTONATED LDHS FOR ENERGY STORAGE

### 3.1 Aqueous battery cathodes

**FIGURE 8:**
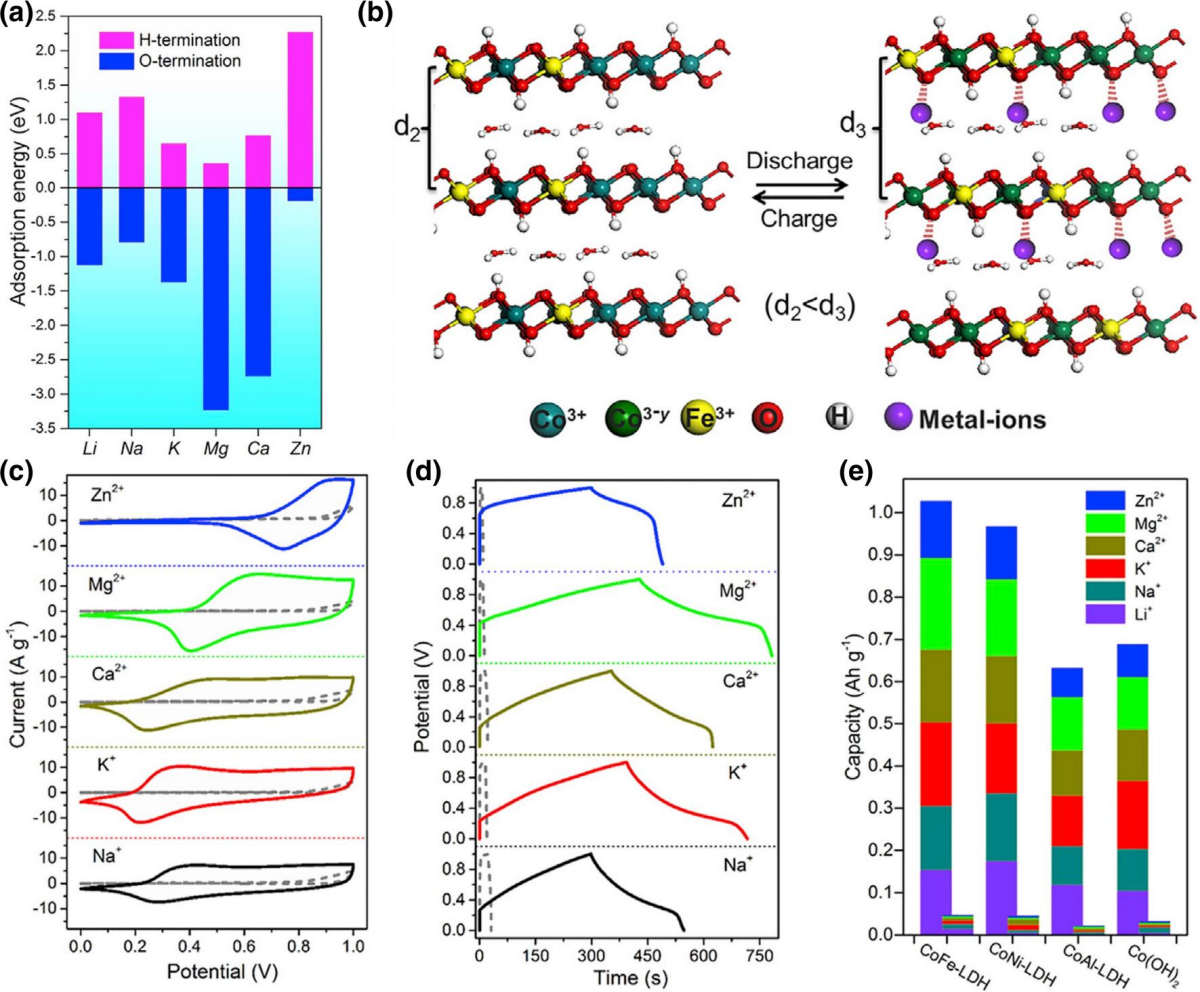
(a) Adsorption energies of metal atoms over OH‐terminated and O‐terminated LDH laminates. (b) Schematic illustration of the structure and composition change of CoFe‐LDH during the ECA process. (c) CV curves of deprotonated CoFe‐LDH and pure CoFe‐LDH in different metal‐salt solutions at a scan rate of 10 mV s^−1^. (d) Corresponding GV curves of deprotonated CoFe‐LDH and pure CoFe‐LDH in different metal‐salt solutions at a current density of 2 A g^−1^. (e) Ion storage properties for the CoFe‐LDH, CoNi‐LDH, CoAl‐LDH, and Co(OH)_2_ electrode before and after deprotonation.^[20]^ *Copyright © 2018 Cell Press.*

Aqueous rechargeable batteries using nonflammable and nontoxic water solvents with abundant multi‐ion cations are promising alternatives for the energy storage field due to their high safety and low cost. The reactions and mass exchange of charge carriers at the electrode can be classified into three types: insertion reactions, conversion reactions, and deposition reactions.^[45]^ Among them, insertion reactions involve the accommodation and release ‘guest’ charge carrier ions into/from ‘host’ materials during redox processes. LDHs, which possess a well‐defined interlayer structure, can in principle be hosts for various anions intercalation. However, the terminal H on LDHs is unfavorable for the adsorption of metal ions and results insufficient active sites for cation accommodation (*i.e.*, repulsive to cations, Figure 8a),^[20]^ thus only limit capacity in neutral solution. In contrast, the deprotonated LDHs with O termination gave negative adsorption energies toward various cations (*i.e.*, Li^+^, Na^+^, K^+^, Ca^2+^, Mg^2+^, and Zn^2+^), indicating the thermodynamically favorable process for cation intercalation Figure 8b. Therefore, the phase transition of LDHs is also important for the electrochemical performance of aqueous rechargeable battery cathode.

We firstly investigated the electrochemical performance of deprotonated LDHs in neutral solution with different cations.^[20]^ As expected, high capacitances of 151, 198, 172, 217, and 136 mAh g^−1^ are obtained by deprotonated CoFe‐LDH for Na^+^, K^+^, Ca^2+^, Mg^2+^, and Zn^2+^ intercalation, which are 27, 33, 34, 43, and 24 times larger than that of pristine CoFe‐LDH respectively (Figure 8c–e). More interestingly, the E_ads_ toward different cations are high relative to their capacity of deprotonated CoFe‐LDH, for example, the ions with lower E_ads_ possess higher specific capacity. The capacity of other deprotonated Co‐based LDH (*i.e.,* CoNi‐LDH, CoAl‐LDH) and Co(OH)_2_ is also dozens of times higher than that of pure LDH without deprotonation. Liu’s group investigated the detailed electrochemical activation (deprotonation) process of CoNi‐LDH and investigated the intercalation performance in Zn rich solution (ZnSO_4_ electrolyte with pH value of 4). The as ‐ prepared deprotonated CoNi‐LDH delivers a high specific capacity of 185 mAh g^−1^ with high discharge voltage of ≈1.61 V versus Zn^2+^/Zn at a current density of 1.2 A g^−1^, which is four times higher than that of the pure CoNi‐LDH electrode (42 mAh g^−1^). Experimental and computational results reveal that a reversible H^+^/Zn^2+^ co‐intercalation mechanism is exhibited for deprotonated CoNi‐LDHs, which is attributed to the lower adsorption energies on O termination in deprotonated LDHs. In contrast, the adsorption energies for H and Zn on terminal H sites are positive (that is 2.41 and 0.94 eV, respectively), indicating the unfavorable intercalation process.^[46]^

**FIGURE 9:**
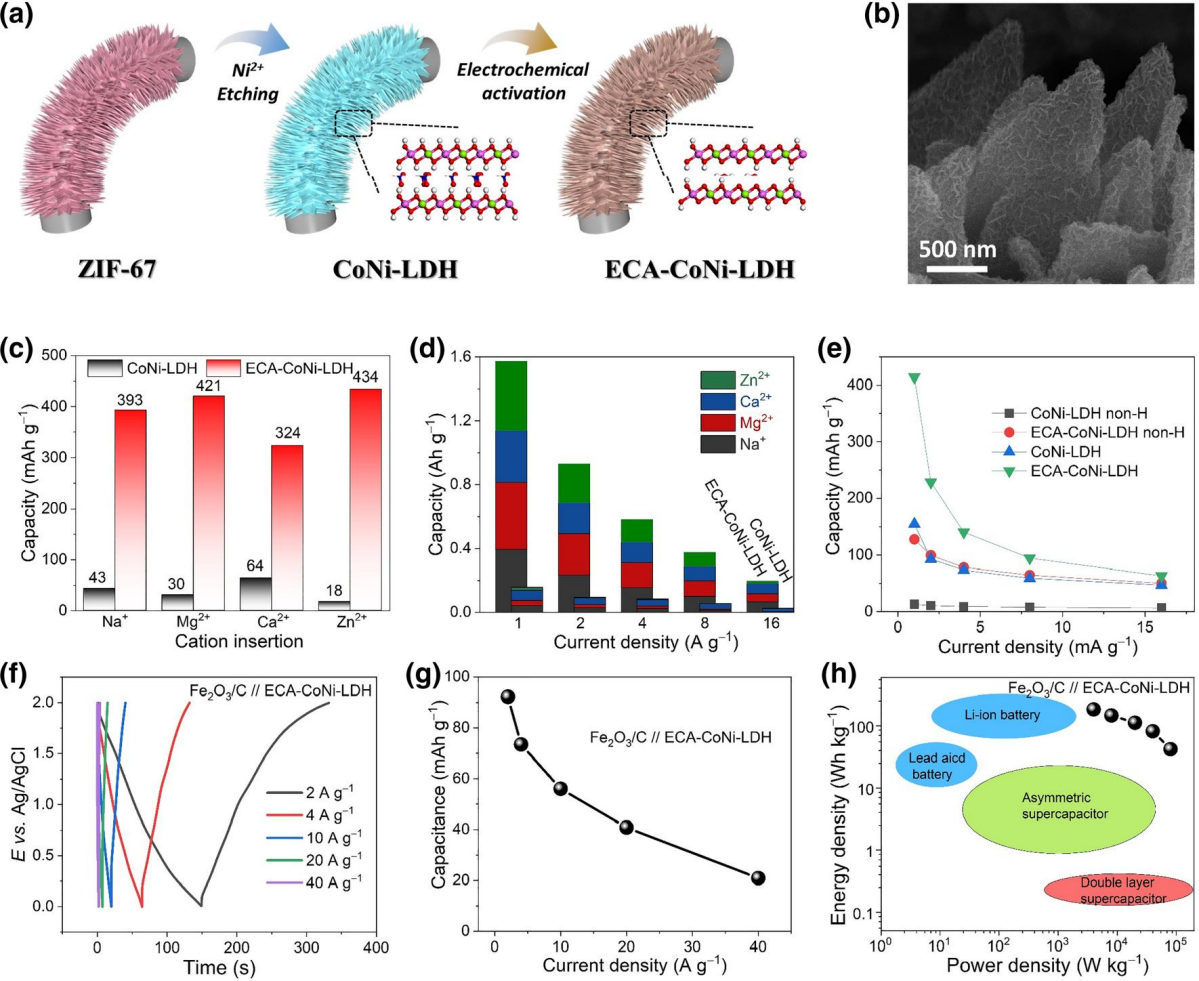
(a) Schematic illustration for the synthesis of deprotonated hierarchical CoNi‐LDH; (b) SEM image of deprotonated hierarchical CoNi‐LDH; (c) the capacities of hierarchical CoNi‐LDH before and after deprotonation process; (d) the capacities of hierarchical CoNi‐LDH at different current density before and after deprotonation process; (e) the capacities of CoNi‐LDH with and without hierarchical structure at different current density before and after deprotonation process; (f) charge and discharge curves of full battery at different current densities; (g) the capacities of fully battery at different current densities; (h) the Ragone plots of the full battery.^[47]^ *Copyright © 2022 Elsevier Inc.*

The deprotonated CoNi‐LDH exhibits higher capacity with a hierarchical structure due to the sufficient exposure of the active sites and satisfactory conductivity. Shao et.al. prepared CoNi‐LDHs nanosheet arrays on carbon nanosheet arrays and formatted 3D hierarchical structures (Figure 9a).^[47]^ This interwoven CoNi‐LDH nanosheet structure not only exhibits large surface area but provides opening diffusion channel to facilitate dispersion and diffusion of the electrolyte (Figure 9b). Remarkably, the hierarchical deprotonated CoNi‐LDH achieves high capacity of 393, 420, 324, 434 mAh g^−1^ for Na^+^, Mg^2+^, Ca^2+^, and Zn^2+^ intercalation at current density of 2 A g^−1^, which is more than 9 times and 3 times higher than that of pure CoNi‐LDH and deprotonated CoNi‐LDH without hierarchical structure (Figure 9c–e). Benefitting from the high excellent performance of hierarchical deprotonated CoNi‐LDH with large potential window, the assembled full battery can be operated within an ultrahigh voltage window of 2 V in neutral aqueous electrolyte (Figure 9f). Moreover, a high energy density of 184.4 Wh kg^−1^ is delivered for the full battery at a power density of 4 Wh kg^−1^, and 41.8 Wh kg^−1^ remained at 80 Wh kg^−1^(Figure 9g,h). The high energy density and power density is comparable with that of commercial Li‐ion batteries.

The deprotonated CoNi‐LDH exhibits high capacity and good rate performance in alkaline solution. Qiu et.al. investigated the deprotonation process and electrochemical performance of CoNi‐LDH in CV measurement. Both the redox peak intensity and the integrated area of the CV curves increased with cycling. After 110 cycles, the CoNi‐LDH converts to deprotonated CoNi‐LDH and the CV curves tend to be stable. The deprotonated CoNi‐LDH electrode displayed a 12‐fold increase in the integrated area of the CV curves after the deprotonation process. The corresponding the specific capacity of deprotonated CoNi‐LDH reaches 545 C g^−1^ at 1 A g^−1^ and 410 C g^−1^ is retained even when the current density increases to 100 A g^−1^.

Ammonium ion batteries using the nonmetal charge carrier NH_4_^+^ exhibit great potential for next‐generation energy storage devices due to their low cost and high safety. Liu *et.al*. fabricated a deprotonated Ni‐Co LDH with an amorphous structure for NH_4_^+^ storage. The deprotonated Ni‐Co LDH exhibited specific capacity (202.4 mAh g^−1^) with a high discharge plateau (0.5 V vs. SCE) in NH_4_^+^, which is higher than that of in K^+^ and Na^+^ based electrolyte.[^48^, ^49^] Spectroscopy results showed that the deprotonated Ni‐Co LDH experienced a NH_4_^+^/H^+^ co‐insertion mechanism.^[50]^.

**FIGURE 10:**
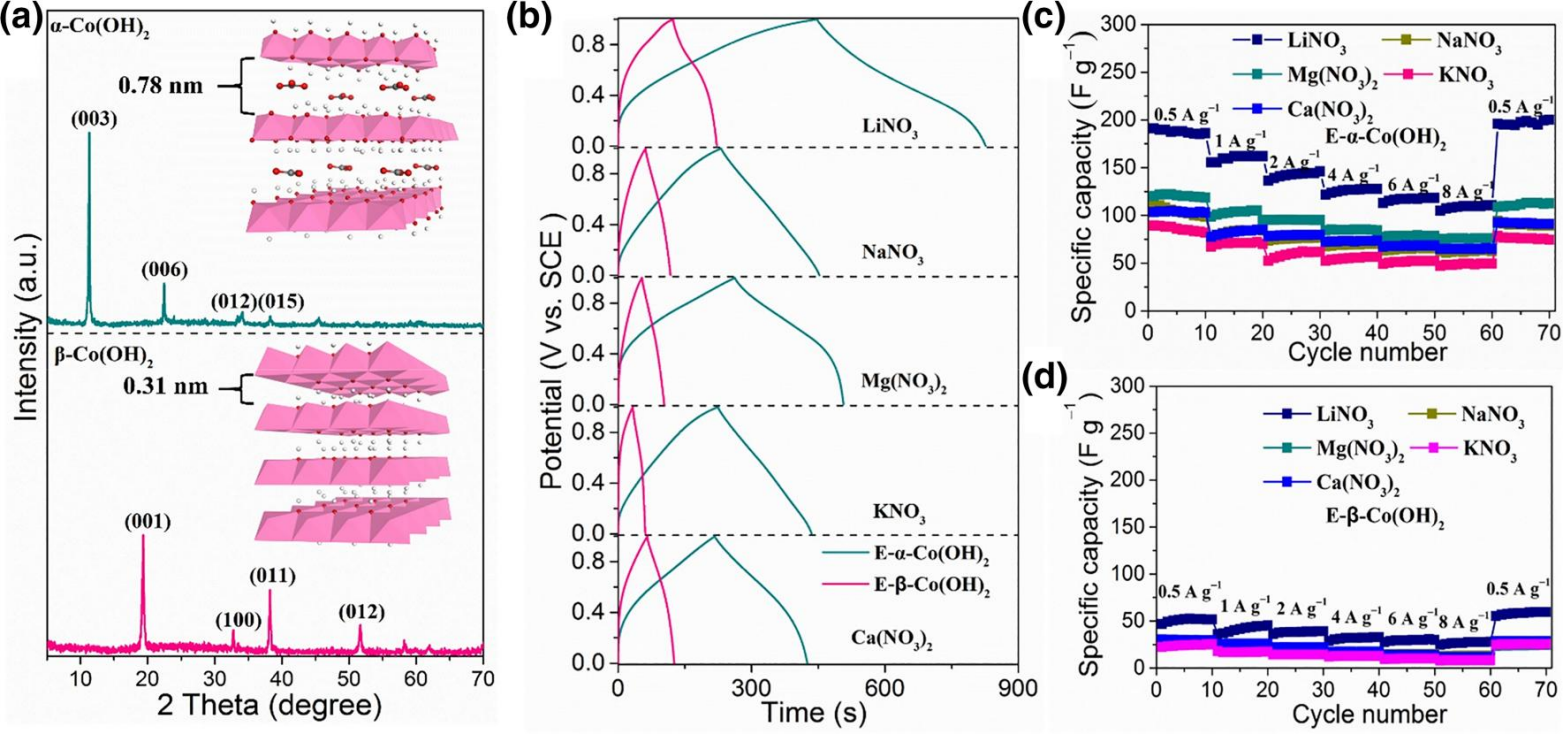
(a) The XRD pattern and corresponding crystal structure of *α*‐Co(OH)_2_ and *β*‐Co(OH)_2_. (b) Charging discharging curve of *α*‐Co(OH)_2_ and *β*‐Co(OH)_2_ with and without deprotonation process. (c) The capacity of deprotonated *α*‐Co(OH)_2_ at different current densities (d) the capacity of deprotonated *β*‐Co(OH)_2_ at different current densities.^[44]^ *Copyright © 2021 Royal Society of Chemistry.*

Other Co‐free‐LDHs were also investigated for the deprotonated process and worked as aqueous battery cathodes. Typically, the deprotonated NiFe‐LDH was fabricated by Wang.^[51]^ A higher potential (1.2 V vs. SCE) is required for NiFe‐LDH than Co based LDH (<0.8 V vs. SCE) for the deprotonation process. The Ni^3+^/Ni^2+^ ratio of the activated NiFe‐LDHs is ca. 2.4, which is two times higher than that of the initial NiFe‐LDH, indicating the successful deprotonation process on the Ni element. The iron element in LDH is robust in the LDH framework. A high capacity of ∼107 mAh g^−1^ is exhibited for activated NiFe‐LDH, which is 50 times than that of the‐NiFe‐LDH electrode. Moreover, the capacity retention of the deprotonated NiFe‐LDH electrode was over 98% after 10,000 cycles. In a recent work, we investigated the deprotonation process and the electrochemical performance of monometallic hydroxide ‐ Co(OH)_2_ with different crystal structures (see Figure 10). The deprotonation process of *α*‐Co(OH)_2_ was similar to that of LDHs. As expected, deprotonated *α*‐Co(OH)_2_ exhibits greatly improved performance for various metal ions (*e.g.,* Li^+^, Na^+^, K^+^, Mg^2+^ and Ca^2+^) with capacitances of 191.0, 112.8, 89.2, 120 and 103.3 F g^−1^. In the contrast, the H is hardly removed on *β*‐Co(OH)_2_ laminate. The “deprotonated” *β*‐Co(OH)_2_ shows relative low capacity in various cation solutions, which is one‐fourth of a quarter of the capacity of deprotonated *α*‐Co(OH)_2_.^[44]^

To date, few host materials (*e.g.,* activation carbon, MXenes, MoS_2_, MoO_*x*_) have worked as a hosts for multiple cation intercalation. Some representative electrode materials and the corresponding electrochemical performances are listed in Table 1. These materials exhibited selective cation intercalation with limited capacity. In contrast, various H vacancies active sites are exposed on deprotonated LDHs for multiple cation intercalation. With the recent advances in deprotonated LDHs in multiple ion intercalation, more novel designs have been achieved. Table 1 summarizes the electrochemical performance of multi‐cations intercalation using fabricated advanced deprotonated LDHs.

**TABLE 1:** The electrochemical performance of deprotonated LDHs and other multiple cation materials.

	Li^+^	Na^+^	K^+^	Mg^2+^	Ca^2+^	Zn^2+^	NH_4_^+^
MoO_*x*_ ^[10]^	132	146	82	150	145	199	‐
MoO_*x*_ ^[10]^	38	32	26	15	26	6	‐
Ti_3_C_2_T_*x*_ ^[7]^	15	12	13	‐	‐	4	‐
MnO_2_ nanobelts^[52]^	49	28	49	‐	‐	‐	‐
AC^[53]^	32	29	‐	‐	‐	‐	‐
MoS_2_ ^[8]^	30	29	28	‐	‐	‐	‐
HOPC^[53]^	89	‐	‐	‐	‐	98	‐
Ni MOF^[13]^	72	69	86	‐	‐	‐	‐
QA COF^[12]^	126	71	‐	‐	‐	‐	‐
VN^[54]^	18	58	‐	‐	‐	‐	‐
Activated carbon^[55]^	64	‐	‐	‐	‐	69	‐
ECA‐CoFe‐LDH^[20]^	121	116	153	175	162	92	‐
H‐ECA‐CoNi‐LDH^[47]^	‐	393	‐	420	324	434	‐
E‐CoNi DH^[48]^	‐	‐	‐	‐	‐	‐	202
SR‐CoFe LDHs^[56]^	‐	‐	‐	‐	‐	‐	168
A‐NiCo DH^[50]^	‐	‐	‐	‐	‐	281	281
H_v_‐Ni_3_Mn_0.7_Fe_0.3_‐LDH^[57]^	‐	‐	‐	‐	‐	328	‐
ECA‐NiFe‐LDHs^[51]^	107	‐	‐	‐	‐	‐	‐
CoNi LDH(v)^[46]^	‐	‐	‐	‐	‐	185	‐
E‐α‐Co(OH)_2_‐P^[58]^	31	25	33	29	‐	‐	‐
E‐α‐Co(OH)_2_‐A^[58]^	78	67	97	80	‐	‐	‐

### 3.2 Metal anodes

**FIGURE 11:**
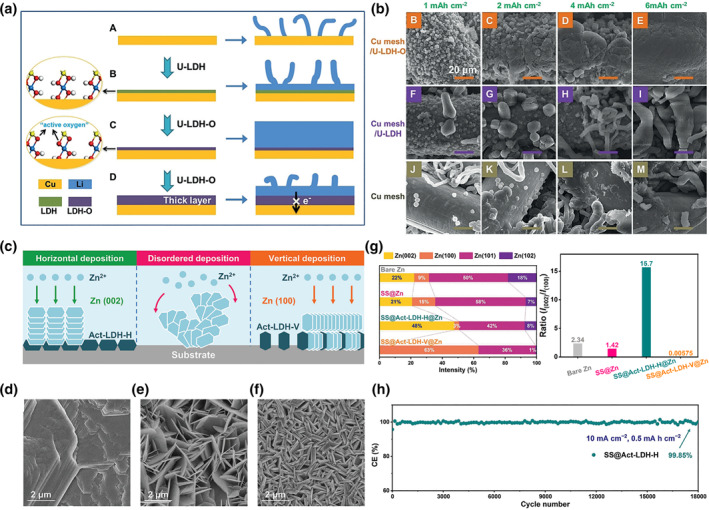
(a) Illustration of Li‐metal growth on Cu substrates, CoFe‐LDH modified Cu substrates, thin deprotonated CoFe‐LDH modified Cu substrates, and thick thin deprotonated CoFe‐LDH modified Cu substrates (b) the SEM image of Li metal plating on Cu substrates, CoFe‐LDH modified Cu substrates, thin deprotonated CoFe‐LDH modified Cu substrates.^[44]^ *Copyright © 2019 Wiley‐VCH GmbH.* (c) Schematic illustration of (002), (100)‐oriented Zn and unoriented Zn plating on deprotonated LDH‐H, deprotonated LDH‐V, and. SEM image of Zn plating on (d) deprotonated LDH‐H, (e) bare stainless steel and (f) deprotonated LDH‐V. (g) The ratio of Zn crystal planes on different plating. (h) CE plots of deprotonated LDH‐H at 10 mA cm^−2^/0.5 mAh cm^−2^.^[63]^ *Copyright © 2023 Wiley‐VCH GmbH.*

Rechargeable metal batteries exhibit high energy densities due to the high theoretical energy density and low electrochemical potential of metallic anodes. For example, Li metal exhibits an ultrahigh theoretical capacity of 3862 mAh g^−1^ and the low electrochemical redox potential of −3.04 V versus SHE. However, the practical application of stable metal batteries is limited by uncontrollably dendrite growth, resulting in low Coulombic efficiency, irreversible capacity‐loss and serious security risks. Constructing nucleophilic materials on the electron collector surface has been proven to be an effective strategy to guide metal growth and suppress metal dendrites. LDHs with terminal H exhibit unfavorable interaction with cations at the molecular level that virtually makes it impossible to use pure LDHs directly for nucleophilic materials. An abundant activated O site is exposed on deprotonate LDH, which boosts the binding between the LDH with cations. The deprotonated LDH can essentially work as nucleation seeds for the homogeneous metal deposition.^[59]^ For example, the coated deprotonated CoFe‐LDH on the Cu mesh inhibited dendrites and improved battery stability (Figure 11a). A coulombic efficiency of 97.3% still remained for deprotonated CoFe‐LDH after 200 cycles, while the CE of pure Cu mesh decayed rapidly to 70.2% after 90 cycles (Figure 11b). Both experimental studies and DFT calculations reveal that the terminal‐O on deprotonated CoFe‐LDH plays a key role in the homogeneous nucleation and plating of Li metal. Moreover, the lithiophilic property of terminal‐O is also related to its coordination environments, where the Co‐O‐Fe structure in deprotonated CoFe‐LDH largely facilitates the Li nucleation.

Meanwhile, regulation orientation of crystal growth is another strategy to express dendrite growth, which can be achieved by reversible epitaxial electrodeposition.^[60]^ In this process, a coherent or semicoherent lattice interface is first formatted at the surface of the substrate with minimal interfacial energy and lattice strain, which enable homoepitaxial metal deposition to create uniform metal coatings with the same orientation. The low lattice mismatch between Zn (002) face and (deprotonated) LDHs exhibits great potential to facilitate heteroepitaxial nucleation.[^61^, ^62^] Recently, Shao ’s group reported a Zn metal anode with uniform (002)‐oriented for highly stable and efficient zinc batteries by directional cation recognition layer – deprotonated LDHs.^[63]^ Ultrathin deprotonated CoFe‐LDH with horizontal (deprotonated LDH‐H) and vertical (deprotonated LDH‐V) arrangement is prepared on stainless steel foil to regulate the crystal orientation (Figure 11c–f). The high crystallographic compatibility (the lattice constants of deprotonated LDH and Zn (002) are 2.787 and 2.667 Å, lattice fitting rate >95.5%) induces the heteroepitaxial Zn nucleate on deprotonated LDH and grows along the direction of the [002] texture. Moreover, the abundant lattice oxygen of deprotonated LDH is also important for reversible epitaxial electrodeposition. The deprotonated LDH oxygen exhibits favorable thermodynamic adsorption behaviors for Zn^2+^ adsorbed, inducing zinc atom homogeneous nucleation. As a result, the Zn metal with an ultrahigh/ultralow facet ratio of (002)/(100) was achieved on deprotonated LDH‐H (15.7) and deprotonated LDH‐V (0.006) (Figure 11g). As expected, a high CE of 99.85% is exhibited for LDH‐H@Zn even after 18,000 cycles at a high current density of 10 mA cm^−2^/0.5 mA cm^−2^. The long duration is more than 10 times longer than that of LDH‐V@Zn or pure Zn foil (Figure 11h).

## 4 CONCLUSIONS AND PERSPECTIVES

In summary, we have systematically introduced the phase transformation of LDH in electrocatalytic water oxidation and energy storage, which undergo similar processes and emerge of deprotonated *γ*‐phase derived from LDHs. In the small potential window (supercapacitor process, before OER), OH^−^ adsorbed on the surface of LDH and promoted breaking the O–H bond on LDH. Proton(s) H in LDH laminate is lost (deprotonation) and converts to MOOH (MOO). In this process, the laminate elements release electron(s) and occurs redox reaction. In a larger potential window (OER process), the LDH converts to MOOH (MOO) with high OER activity and electrocatalytic water oxidation to release O_2_. Additionally, the recent development of deprotonated LDHs in aqueous and metal batteries reviewed. Various deprotonated LDHs (Co‐based, Ni‐based and Mn‐based) with O termination exhibit negative adsorption energies toward different cations (*e.g.* Li^+^, Na^+^, Zn^2+^ etc.), which exhibit high capacity and long cycling life for aqueous battery cathodes. Moreover, benefitting from the high interaction between cation and deprotonated LDH, deprotonated LDH can be worked as nucleation seeds for the homogeneous metal deposition.

To date, for the development of deprotonated LDHs in energy storage, challenges remain for performance optimization in terms of the deprotonated mechanism and electrochemical performance. (i) The electrochemical performance of deprotonated LDH aqueous battery cathodes is highly influenced by the deprotonation process of LDHs. LDHs with different laminate elements exhibit the various interactions between OH, resulting in the deprotonation process for different LDHs being normally worlds apart. The detailed deprotonation process of LDHs needs further investigation by more advanced characterization technology. (ii) Currently, only a few deprotonated LDH aqueous battery cathodes work as aqueous battery cathodes, including Co‐based, Ni‐based and Mn‐based LDHs. To achieve high performance of aqueous battery full cells, more deprotonated LDH electrode materials with higher capacity and higher potential window are crucial to be developed. (iii) Various cations have been proved to be intercalated into deprotonated LDH cathodes in aqueous solution. However, the aqueous electrolyte is highly limited by the narrow potential window. Resembling the organic electrolyte (e.g. PC, EC/DME) can be adopted to deprotonate LDHs based batteries to extend the operation voltage window, which will offer more choices of deprotonated LDHs in the expanded voltage window. (iv) The deprotonated CoFe‐LDH has been proved as a nucleophilic material to guide Li and Zn metal growth and suppress dendrites. More deprotonated LDH electrode materials with different structures should be investigated for the uniform metal deposition. Moreover, the deprotonated LDHs are suggested to extend for other metal anodes with dendrite suppression.

## CONFLICT OF INTEREST STATEMENT

The authors declare no conflicts of interest.
